# Essential medicines for breast cancer in low and middle income countries

**DOI:** 10.1186/s12885-015-1583-4

**Published:** 2015-08-18

**Authors:** Y. T. Bazargani, A. de Boer, J. H. M. Schellens, H. G. M. Leufkens, Aukje K. Mantel-Teeuwisse

**Affiliations:** 1 Division of Pharmacoepidemiology and Clinical Pharmacology, Utrecht Institute for Pharmaceutical Sciences, Utrecht University, David de Wied building, Universiteitsweg 99, 3584 CG Utrecht, The Netherlands; 2 Division of Clinical Pharmacology, The Netherlands Cancer Institute, Amsterdam, The Netherlands

## Abstract

**Background:**

Breast cancer is the most common type of cancer among women worldwide. In low and middle-income countries (LMICs), appropriate selection of medicines on national essential medicines lists (NEMLs) is a first step towards adequate access to treatment. We studied selection of systemic treatments for breast cancer on NEMLs and assessed its alignment with treatment guidelines for different types of early and advanced breast cancer. Furthermore, influence of country characteristics on the selection was investigated.

**Method:**

NEMLs from 75 LMICs were studied for inclusion of all components of therapy in each stage of breast cancer according to international consensus guidelines. The results were then grouped by income level, WHO region and the NEMLs’ release date. Non parametric tests were used for statistical analysis.

**Results:**

Unlike HER2-targeted therapies (<10 %), aromatase inhibitors (12 %) and taxanes (28 %); tamoxifen and first generation chemotherapeutic regimens (e.g., anthracycline-based regimens) were frequently found in the NEMLs (71–78 %). Consequently, all components of treatment for “Luminal A” early breast cancer and non HER2 overexpressed advanced breast cancer were found on the NEMLs of over 70 % of countries. However, 40 % of the low income countries did not have all the components of therapy for any type of early breast cancer in their NEMLs, and adequate treatment of HER2 overexpressed breast cancer was hardly possible with the current selections. Recent NEMLs were more aligned with the guidelines (p < 0.05). Eastern Mediterranean and African regions less frequently incorporated all components of breast cancer treatment in their NEMLs.

**Conclusion:**

Alignment of selection with guidelines’ recommendations was inconsistent for different types of early and advanced breast cancer in NEMLs. Regular updates and more attention to clinical guidelines is therefore recommended.

**Electronic supplementary material:**

The online version of this article (doi:10.1186/s12885-015-1583-4) contains supplementary material, which is available to authorized users.

## Background

Breast cancer is the leading cause of death among women in both developed and developing countries [1]. Substantial progress has been made in the past decades in early detection, screening and treatment of breast cancer. This has resulted in 5-year survival rates of approximately 80 %, 60 % and 40 % for high, middle and low income countries, respectively [2]. Comprehensive national cancer control plans to fight (breast) cancer may consist of prevention, screening and early detection, diagnosis, treatment (surgery, radiotherapy and systemic therapy) and palliative care [3]. Not necessarily all the components of a comprehensive national cancer control plan exist in every low or middle income country (LMIC). In some cases existence and accessibility of facilities for surgery and radiotherapy have even been questioned [4–6].

Little is known about global access to systemic therapy as a part of the treatment of breast cancer. Many international guidelines have been published including guidelines adjusted for resource constrained countries or geographical regions [5–12]. However, availability of recommended therapies according to the guidelines has hardly ever been evaluated although sporadic reports regarding low availability of human epidermal growth factor receptor type 2 (HER2)- targeted therapies in LMICs have been published [13].

Selection of appropriate medication for breast cancer on national essential medicines lists (NEMLs) is an initial step in achieving adequate access to pharmacological treatment in LMICs. Essential medicines are those that satisfy the priority health care needs of the population [14]. They are selected with due regard to disease prevalence, evidence on efficacy and safety, and comparative cost-effectiveness and have an established role in public procurement or reimbursement of medicines in the majority of LMICs. Over 90 % of surveyed LMICs are reported to use their NEML for public procurement of medicines [14]. Consequently, being listed as essential medicine can be seen as a prerequisite for access to a medicine in clinical practice, particularly in the public sector of LMICs where the majority of patients would primarily seek their treatment.

Selection of essential medicines for oncology is suboptimal for newer therapies but more strikingly for conventional therapies and in particular for hormonal therapies across LMICs [15]. As the latter group of medicines plays a pivotal role in breast cancer treatment, we thoroughly studied available NEMLs to assess diversity in selection of breast cancer medicines across LMICs. Besides, we aimed to assess the extent to which these selected essential medicines would allow treatment of different stages of breast cancer according to international treatment guidelines. The influences of country income level, geographic region and year of update of the NEML on the selection were also explored.

## Methods

### Data collection and classification

#### Essential medicines lists

NEMLs from LMICs were obtained in May 2013 from the “WHO database of essential medicine lists and formularies” [16]. The latest available update of the NEMLs was considered for each country. Countries with a NEML dated prior to 2005 were excluded (n = 6). Since the WHO has recommended countries to periodically update their NEMLs, this measure was taken to ensure that only dynamic lists were considered for this study [14]. In China, provincial EMLs were deemed eligible and were added to make an EML list, instead of the NEML, since no essential oncology medicines were found on the NEML of China [17]. Eventually, 75 countries were included in the analysis which were representative of the low and middle income levels across all WHO regions (see Additional file 1: Annex 1).

Medicines were included in the study if they were categorized as oncology medicines in the NEML (or equivalent terms in different NEMLs or languages). Palliative and supportive therapies and medicines for management of side effects and complications were excluded. Of the remaining medicines, only those which were recommended for breast cancer according to international guidelines (see below) were included. Medicines used for breast cancer are generally categorized into three different classes, namely chemotherapeutic agents, endocrine therapy agents and HER2-targeted therapies. Chemotherapeutic agents have a non-selective cytotoxicity and are indicated in different types of malignancies. Endocrine therapy agents play a crucial role in treatment of hormone receptor (estrogen and/or progesterone) positive breast cancer patients [18]. HER2-targeted therapies are shown to increase overall survival in patients with HER2-overexpressing tumors through blockage of extracellular or intracellular components of the HER2 protein [19, 20].

#### Therapeutic guidelines

PubMed was searched to obtain the most recent updates (at the time of study, i.e., July 2013)) of evidence based international consensus guidelines for different stages of breast cancer. Precedence was given to guidelines designed for LMICs when various guidelines were available. Eventually the guidelines were classified into two main groups: (1) Guidelines in which the consensus was based on different types of disease or tumor [7, 8, 11] and (2) Guidelines in which the consensus was formulated for different levels of care (based on available resources and services) [4–6, 9, 10, 12, 21]. These latter guidelines were mainly based on the Breast Health Global Initiative (BHGI) consensus for LMICs modified for implementation in different situations (e.g., different income levels of countries or different regions). As the initial interest of the current study was to investigate which stages of disease and which tumor types can be treated with the selected medicines, the first group of guidelines was selected for the final analyses (namely: St. Gallen International Expert Consensus on the Primary Therapy of Early Breast Cancer [7] and 1st International consensus guidelines for advanced breast cancer (ABC 1) [8]).

Different subtypes of early and advanced breast cancer -according to the guidelines- are described in Table 1. For each type of breast cancer, the necessary components of treatment regimens were identified based on the aforementioned guidelines. Full details of all components for treatment of breast cancer are given in Additional file 1: Annex 2.

**Table 1:** Different subtypes of early and advanced breast cancer according to the selected guidelines (7, 8)

Type of breast cancer	Subtypes	Descriptions
Early breast cancer^†^	Luminal A	HR+;HER2-; Ki-67 low
	Luminal B	HR+;HER2-; Ki-67 high
	Non luminal	HR+;HER2+; Ki-67 low
	Triple negative (ductal)	HR absent; HER2 absent
	Special histological types	HER2 absent; endocrine responsive or non-responsive
Advanced breast cancer^††^	HR +/HER2-	
	HR+/HER2+	
	HR-/HER2+	
	HR-/HER2-	

*HR+* = ER and/or PR positive tumor, *HR-* = ER and/or PR negative tumor, *HER2* human epidermal growth factor receptor type 2 oncogene, *HER2+* = HER2 over expressed or amplified, *HER2-* = not HER2+, *Ki-67* a marker of cell proliferation

^†^Early breast cancer: Breast cancer that has not spread beyond the breast or the axillary lymph nodes. This includes ductal carcinoma in situ and stage I, stage IIA, stage IIB, and stage IIIA breast cancers. *(Reference: National Cancer Institute;* http://www.cancer.gov/dictionary?cdrid=446564*)*

^††^Advanced breast cancer: Breast cancer that has spread locally in the area of the breast (locally advanced breast cancer) or to distant organs and tissues (metastatic breast cancer), it includes: •Stage III: the cancer has either extensively spread to lymph nodes and/or other tissue in the area of the breast, but not to distant sites in the body •Stage IV: the cancer has spread to distant sites of the body, such as the liver, lungs, bones, brain, and/or other sites (*Reference: advanced breast cancer community*; http://www.advancedbreastcancercommunity.org/advanced-breast-cancer/defining-advanced-breast-cancer.html)

^†,††^: Early breast cancer is classified based on clinicopathological criteria namely detection of the estrogen receptor, progesterone receptor, over expression or amplification of the HER2 oncogene and Ki-67 labeling index (a marker of cell proliferation). Advanced breast cancer is classified in a similar way except for the Ki-67 labeling index which has no role in this classification

*(Reference: a) Goldhirsch A, et al. Ann Oncol 2011 Aug;22(8):1736–1747. b) Cardoso F, et al. Breast 2012 Jun;21(3):242–252.)*

#### Other sources of data

Data on geographic regions and income levels were obtained from the WHO and the World Bank, respectively [22, 23].

### Data analysis

First, the frequency of inclusion of breast cancer medicines and combinations (chemotherapeutic or endocrine therapy) as recommended by the international guidelines in different NEMLs were calculated. Then the NEMLs were assessed to see if all components of a particular treatment regimen as mentioned in the therapeutic guidelines could be collectively found for each stage of disease and each type of tumor. In all cases, only classes of medicines - as recommended by the guidelines - were considered, regardless of the number of medicines within each class being designated as essential medicine. The proportion of countries which selected a complete therapeutic regimen was then calculated for each type and stage of disease. Data were subsequently stratified and analyzed according to different income levels and WHO regions. Moreover, in order to compare the extent of compliance with the international guidelines between newer and older NEMLs, the NEMLs released in 2009 and afterwards were compared with the ones published prior to 2009.

When the frequency or percentage of inclusion of treatments in the NEMLs for various types of breast cancer was compared between different clusters of countries, non-parametric tests were used to investigate the differences among groups, namely the Kruskal Wallis test for geographic regions and income levels as well as the Chi square test for recent versus older NEMLs. All statistical analyses were conducted using SPSS software, version 19.

Research ethics statement as requested by the journal was not applicable to our study. All the data used in the manuscript is freely available.

## Results

### Selection of essential medicines for breast cancer

Overall, 84 % and 74 % of the studied countries had at least one chemotherapeutic and one hormonal agent for breast cancer, respectively. Slightly fewer than 10 % of the countries had a HER2-targeted therapy as essential medicine.

**Fig. 1:**
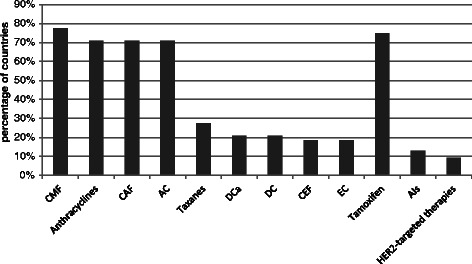
Inclusion of main chemotherapeutic and hormonal therapy regimens in NEMLs (n = 75). CMF: cyclophosphamide, methotrexate and fluorouracil; CAF: cyclophosphamide, doxorubicin (adriamycin), fluorouracil, AC: doxorubicin and cyclophosphamide; EC: epirubicine and cyclophosphamide; CEF: cyclophosphamide, epirubicine and fluorouracil; DCa: docetaxel and carboplatin; DC: docetaxel and cyclophosphamide; AIs: Aromatase Inhibitors

Figure 1 shows the inclusion of the main chemotherapeutic and hormonal regimens for the treatment of breast cancer according to the international guidelines in the NEMLs. Tamoxifen, anthracylines, CMF (cyclophosphamide, methotrexate and fluorouracil), CAF (cyclophosphamide, doxorubicin and fluorouracil) and AC (doxorubicin and cyclophosphamide) were well represented with inclusion in more than 70 % of the NEMLs as opposed to inclusion in below 30 % for all other main regimens.

A more detailed overview of inclusion of individual chemotherapeutic and hormonal agents is illustrated in Additional file 1: Annex 3. Cyclophosphamide, methotrexate, fluorouracil and tamoxifen were found in over 75 % of the NEMLs, and doxorubicin and vinblastine in over 50 %. Of the HER2-targeted therapies, lapatinib was completely absent and trastuzumab was selected in less than 10 % of the NEMLs.

### Selection of treatment regimens for different breast cancer stages

#### Early breast cancer

In three out of four of the studied countries all components for treatment of “Luminal A” type breast cancer were selected as essential medicines. One third of the countries also had all components for the treatment of the “triple negative” type. One in four countries had all components for treatment of four different types including “Luminal B (HER2-)” and “Special histological types” of early breast cancer in addition to the former two types. All treatment components (collectively) for HER2+ tumors were only found in less than 10 % of the NEMLs.

**Fig. 2:**
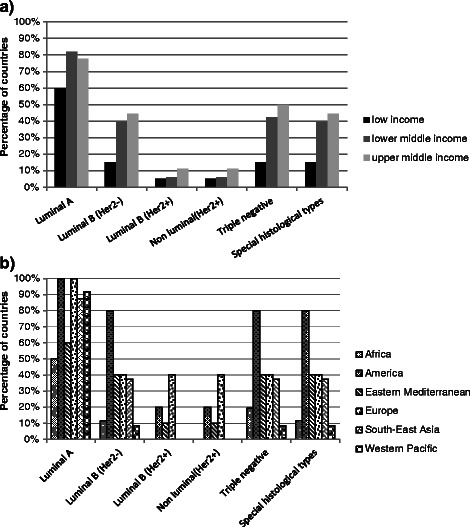
**a** Inclusion of all components of treatment for different types of early breast cancer tumors in the NEMLs across different income levels number of countries: Low income n = 20; Lower middle income n = 33; Upper middle income n = 18. **b** Inclusion of all components of treatment for different types of early breast cancer tumors in the NEMLs across different WHO regions number of countries: Africa n = 26; America n = 14; Eastern Mediterranean n = 12; Europe n = 5; South-East Asia n = 8; Western Pacific n = 12

Figure 2a shows that inclusion of essential medicines was constantly more aligned with the therapeutic guidelines in middle income countries compared to low income countries (p = 0.047). Forty percent of the low income countries did not have all therapy components for any type of early breast cancer.

There was a significant difference across regions in the proportion of countries which had all treatment components for different types of early breast cancer in their NEMLs (p < 0.001). While over 80 % of the American countries included all therapy components for all types of early breast cancer (except for HER2 overexpressed tumors), over 40 % of the countries in the Eastern Mediterranean and African regions did not have all treatment components for any subtype (Fig. 2b).

Across the different types of early breast cancer, newer NEMLs had more frequently incorporated all treatment components compared to the older NEMLs. However, the difference was only statistically significant for “Luminal A” type (87 % for newer NEMLs vs. 62 % for older NEMLs, p = 0.033)

#### Advanced breast cancer

All components for treatment of two types, namely HR-/ HER2- and HR+/ HER2- could be found in over 70 % of NEMLs. In contrast, all components for therapy of HER2+ tumors (regardless of HR status) were found in less than 10 % of the NEMLs studied.

**Fig. 3:**
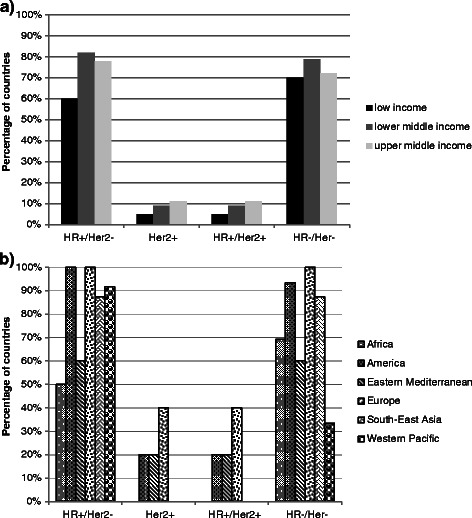
**a** Inclusion of all components of treatment for different types of advanced breast cancer in the NEMLs across different income levels number of countries: Low income n = 20; Lower middle income n = 33; Upper middle income n = 18. **b** Inclusion of all components of treatment for different types of advanced breast cancer in the NEMLs across different WHO regions number of countries: Africa n = 26; America n = 14; Eastern Mediterranean n = 12; Europe n = 5; South-East Asia n = 8; Western Pacific n = 12

The proportion of countries in which all components of 1st line therapy were selected in the NEMLs for different types of advanced breast cancer was not significantly different across the 3 income level categories (p = 0.410). Over half of the countries in all different income levels had all components for 1st line therapy of HR+/HER2- and HR-/ HER2- types of advanced breast cancer as essential medicines (Fig. 3a).

However, the proportion varied significantly across regions (p = 0.017). Above 85 % of the countries in the regions of the Americas, Europe and South-East Asia had all components for therapies of advanced breast cancer (except HER2+), while -on average- 40 % of the countries in the Eastern Mediterranean region and Africa did not have those (Fig. 3b). Unlike in other regions, countries in the Western Pacific region included all treatment components for HR-/HER2- breast cancer treatment less frequently than for HR+/HER2- treatment (33 % vs. 92 %).

Across all different tumor types, newer NEMLs had more frequently incorporated all components of treatments compared to the older ones. The difference was only statistically significant for HR+/HER2- and HR-/HER2- tumor types (87 % and 87 % for newer NEMLs vs. 62 % and 54 % for older NEMLs, p = 0.033 and 0.005, respectively)

Three out of four countries had all components for 2nd line treatment of HR+/HER2- type advanced breast cancer as essential medicines. All components for 2nd line therapy for HR+/HER2+ type of advanced breast cancer were not found in any of the NEMLs.

The differences observed across income level categories and WHO regions and between newer and older NEMLs were almost identical to the first line treatment for HR+ and HER2+ types.

## Discussion

Despite substantial progress made in its treatment possibilities, breast cancer survival is still poor in LMICs. This might be due to lack of access to different components of care including systemic therapy. Selection of essential medicines was explored in this study as a prerequisite of access to medicines. First generation chemotherapies were frequently found in the NEMLs of the studied countries (>70 %). Endocrine therapy was also well represented with tamoxifen being included in 75 % of the NEMLs, whereas treatment of HER2 overexpressed tumors (in both early and advanced stages) was hardly possible with the selected essential medicines. Except for luminal A breast cancer, selection of essential medicines did not allow treatment of early breast cancer subtypes in many LMICs. Guideline- recommended treatments were less frequently included in the NEMLs of low income countries than in middle income countries. In advanced breast cancer, all components of therapy (except for HER2-targeted therapies) were included in over half of the NEMLs with no significant differences across income levels. Compared to the other regions, the Eastern Mediterranean and African regions less frequently incorporated full breast cancer treatment components for both early and advanced breast cancer. Across all different breast cancer types, newer NEMLs were more frequently aligned with clinical guidelines.

Considering the fact that breast cancer is the most burdensome type of cancer among women, the selection of therapies for different disease stages is suboptimal. Treatments for late stages were more frequently selected as essential medicines compared to (several) early stages. In early breast cancer, treatment was mainly absent for luminal B (HER2-) in low income countries and for triple negative and special histological types in low and lower middle income countries. This finding can be interpreted as a rational response to health care priorities of resource-constrained countries. Due to lack of screening programs, diagnostic facilities, routine checkups and cultural barriers for educating women for self-examinations, breast cancer is usually diagnosed at late stages in low income countries [5, 12, 24, 25]. In addition, low income countries have to prioritize their decisions, owing to their very limited health care budgets which hardly exceeded an average of US$30 per capita in 2011 [26].

The choice of chemotherapeutic regimens - which were included to a relatively high extent in the NEMLs - might have been heavily influenced by the WHO model list of essential medicines. This can explain the absence of epirubicine based regimens in the majority of the NEMLs studied, since epirubicine is absent in the WHO model list [27, 28]. The only major deviation from the WHO model list are taxanes which have a low rate of inclusion in the NEMLs despite being listed by the WHO. This might be attributable to their late inclusion in the WHO model list in 2011; it needs additional time before medicines appear on the majority of NEMLs. Besides, in the years prior to 2011, affordability of taxanes was of concern for healthcare systems in LMICs [29–31].

Inclusion of aromatase inhibitors’ (AIs) was low compared to tamoxifen. The cost of AIs might have hampered their selection despite their therapeutic benefits [4]. In recent years patent protection of some AIs expired and a price decline was already observed [32]. Subsequently LMICs may consider AIs for inclusion on their NEML as the affordability concern is fading away. In addition, a careful trade off may need to be made by policymakers in view of the limited resources, whether priority should be given to additional hormonal therapies. HR+ breast cancer corresponds to the majority of breast cancer cases and thus has a crucial role in treatment. However the magnitude of benefit gained by substitution of AIs with Tamoxifen in post-menopausal patients may need to be compared with that of adding a HER-2 targeted therapy to the current practice in a smaller group of patients.

A very low rate of inclusion of “all components” of treatment for HER2 overexpressed types of breast cancer (which constitute nearly one fourth of all cases [33]) in the NEMLs in both stages was evident. This was mainly due to the absence of HER2-targeted therapies while patients may still benefit from favorable effects of chemotherapy regimens. For inclusion of HER2-targeted therapies a great deal of controversy should always be addressed, even in developed countries. The percentage of patients with HER2 overexpression may vary across LMICs and the information is lacking in many of these jurisdictions. The massive economic burden incurred to health care systems, cost effectiveness concerns and recent reports of resistance against therapy are examples of these controversies [34–37]. Besides, molecular diagnostic tests for appropriate patient selection are often not integrated in routine daily practice in LMICs due to the lack of resources and equipment [38]. Trastuzumab was first approved over 1.5 decade ago, and its patent will expire soon which will provide an opportunity for better access to the medicine for patients in LMICs [39]. Due attention to the rational use of trastuzumab will remain a concern even when inclusion in the NEMLs will become feasible.

Rank of breast cancer (in terms of incidence and mortality among all cancer types in women) in eastern Mediterranean and African regions is comparable with the global pattern [1]. Other priorities in those health care systems might have hindered inclusion of breast cancer medicines in the NEMLs in those regions. The main common diseases to tackle in those regions are lower respiratory infections, diarrheal disease, preterm birth complications and tuberculosis as well as malaria and HIV/AIDS for the African and cardiovascular disease for the Eastern Mediterranean regions [40, 41].

This study has some limitations. While the most recent breast cancer guidelines -at the time of survey -were studied, the majority of the NEMLs investigated were dated prior to these guidelines. However, the latest available update of a NEML should be considered valid until revision. Our analysis was based on international consensus guidelines while clinical practice might vary across and within the studied countries. Treatment of secondary metastasis (e.g., treatment of bone and brain metastasis), palliative care and medicines for management of adverse events and side effects were not included in our study, despite their essential role in the course of treatment. In addition, as previously mentioned, pharmacotherapy is only one component of breast cancer care. In the entire procedure of treatment of breast cancer a substantial degree of disparity in access and quality of care might be observed [42]. For example in some countries in Africa estrogen receptor identification is not yet routinely accessible [43]. A systemic approach to explore availability of all contributing elements of care for breast cancer, would be a next step for further study.

Although guidelines for management of breast cancer in LMICs have been published in literature, to our knowledge this study is the first global study attempting to explore decisions made for selection of systemic therapy of breast cancer in LMICs, which in turn may have direct implications for the availability of medicines for treatment of breast cancer. Previous research showed that in general essential medicines selected on NEMLs were more available than those not selected as essential medicines, highlighting the importance of adequate selection to ensure optimal access [44]. It is of prime priority to conduct direct availability studies on oncology medicines in LMICs to confirm these findings.

## Conclusion

First generation chemotherapeutic agents and tamoxifen were selected as essential medicines for breast cancer treatment on NEMLs by the vast majority of LMICs. HER2-targeted therapies for treatment of HER2+ tumors and taxanes were notably absent in the majority of NEMLs. Attention to treatment guidelines can assist countries to select essential medicines, which allow treatment of more types and stages of breast cancer.

## Additional file

Additional file 1: **Annex 1**. Overview of LMICs included in the study (World Bank income level, WHO region, year of NEML publication). **Annex 2**: International consensus guidelines for breast cancer treatment. **Annex 3**: Frequency of inclusion of oncology medicines indicated for breast cancer in the NEMLs studied. (PDF 449 kb)

## Abbreviations

LMICs: Low and middle-income countries
NEMLs: National essential medicines lists
HER2: Human epidermal growth factor receptor type 2

## References

[1] American Cancer Society. Global Cancer Facts & Figures. 2011; Available at: http://www.cancer.org/acs/groups/content/@epidemiologysurveilance/documents/document/acspc-027766.pdf. Accessed 12/20, 2012.

[2] World Health Organization. Breast cancer: prevention and control. 2013; Available at: http://www.who.int/cancer/detection/breastcancer/en/index1.html. Accessed 7/25, 2013.

[3] World Health Organization. National cancer control programmes. 2013; Available at: http://www.who.int/cancer/nccp/en/. Accessed 7/25, 2013.

[4] Eniu A, Carlson RW, El Saghir NS, Bines J, Bese NS, Vorobiof D (2008). Guideline implementation for breast healthcare in low- and middle-income countries: treatment resource allocation. Cancer.

[5] El Saghir NS, Adebamowo CA, Anderson BO, Carlson RW, Bird PA, Corbex M (2011). Breast cancer management in low resource countries (LRCs): consensus statement from the Breast Health Global Initiative. Breast.

[6] Anderson BO, Cazap E, El Saghir NS, Yip CH, Khaled HM, Otero IV (2011). Optimisation of breast cancer management in low-resource and middle-resource countries: executive summary of the Breast Health Global Initiative consensus, 2010. Lancet Oncol.

[7] Goldhirsch A, Wood WC, Coates AS, Gelber RD, Thurlimann B, Senn HJ (2011). Strategies for subtypes--dealing with the diversity of breast cancer: highlights of the St. Gallen International Expert Consensus on the Primary Therapy of Early Breast Cancer 2011. Ann Oncol.

[8] Cardoso F, Costa A, Norton L, Cameron D, Cufer T, Fallowfield L (2012). 1st International consensus guidelines for advanced breast cancer (ABC 1). Breast.

[9] Anderson BO, Shyyan R, Eniu A, Smith RA, Yip CH, Bese NS (2006). Breast cancer in limited-resource countries: an overview of the Breast Health Global Initiative 2005 guidelines. Breast J.

[10] El Saghir NS, Eniu A, Carlson RW, Aziz Z, Vorobiof D, Hortobagyi GN (2008). Locally advanced breast cancer: treatment guideline implementation with particular attention to low- and middle-income countries. Cancer.

[11] Anderson BO, Cazap E (2009). Breast health global initiative (BHGI) outline for program development in Latin America. Salud Publica Mex.

[12] Yip CH, Cazap E, Anderson BO, Bright KL, Caleffi M, Cardoso F (2011). Breast cancer management in middle-resource countries (MRCs): consensus statement from the Breast Health Global Initiative. Breast.

[13] Goss PE, Lee BL, Badovinac-Crnjevic T, Strasser-Weippl K, Chavarri-Guerra Y, St Louis J (2013). Planning cancer control in Latin America and the Caribbean. Lancet Oncol.

[14] van den Ham R, Bero L, Laing R. The World Medicines Situation 2011; selection of essential medicines. Available at: http://apps.who.int/medicinedocs/en/m/abstract/Js18770en/. Accessed 12/4, 2012.

[15] Bazargani YT, de Boer A, Schellens JH, Leufkens HG, Mantel-Teeuwisse AK (2014). Selection of oncology medicines in low- and middle-income countries. Ann Oncol.

[16] World health Organization. Essential Medicines Lists and Formularies. Available at: http://www.who.int/selection_medicines/country_lists/en/index.html. Accessed 11/23, 2012.

[17] Wang L, Ma E, Xu W. Comparative Analysis of China National & Twenty-two Selected Provincial Essential Medicine Lists to the WHO 2011 Model List. Available at: http://apps.who.int/medicinedocs/en/m/abstract/Js18851en/. Accessed 11/23, 2012.

[18] Sheri A, Johnston S (2013). New developments and future directions in systemic therapy. Clin Oncol (R Coll Radiol).

[19] Slamon DJ, Leyland-Jones B, Shak S, Fuchs H, Paton V, Bajamonde A (2001). Use of chemotherapy plus a monoclonal antibody against HER2 for metastatic breast cancer that overexpresses HER2. N Engl J Med.

[20] Slamon D, Eiermann W, Robert N, Pienkowski T, Martin M, Press M (2011). Adjuvant trastuzumab in HER2-positive breast cancer. N Engl J Med.

[21] Wong NS, Anderson BO, Khoo KS, Ang PT, Yip CH, Lu YS (2009). Management of HER2-positive breast cancer in Asia: consensus statement from the Asian Oncology Summit 2009. Lancet Oncol.

[22] World health Organization. WHO geographic regions. Available at: http://www.who.int/about/regions/en/index.html. Accessed 11/23, 2012.

[23] The World Bank. World Bank classification of countries. Available at: http://data.worldbank.org/indicator/NY.GNP.PCAP.CD. Accessed 11/23, 2012.

[24] Coughlin SS, Ekwueme DU (2009). Breast cancer as a global health concern. Cancer Epidemiol.

[25] Brower V (2011). Developing nations face challenges as breast cancer rises. J Natl Cancer Inst.

[26] The World Bank. Health expenditure per capita (current US$). 2014; Available at: http://data.worldbank.org/indicator/SH.XPD.PCAP/countries/XM?display=graph. Accessed 02/11, 2014.

[27] Chavarri-Guerra Y, Villarreal-Garza C, Liedke PE, Knaul F, Mohar A, Finkelstein DM (2012). Breast cancer in Mexico: a growing challenge to health and the health system. Lancet Oncol.

[28] Lee BL, Liedke PE, Barrios CH, Simon SD, Finkelstein DM, Goss PE (2012). Breast cancer in Brazil: present status and future goals. Lancet Oncol.

[29] Griffin S, Dunn G, Palmer S, Macfarlane K, Brent S, Dyker A (2009). The use of paclitaxel in the management of early stage breast cancer. Health Technol Assess.

[30] Chilcott J, Lloyd Jones M, Wilkinson A (2009). Docetaxel for the adjuvant treatment of early node-positive breast cancer: a single technology appraisal. Health Technol Assess.

[31] Lopes GL (2013). Cost comparison and economic implications of commonly used originator and generic chemotherapy drugs in India. Ann Oncol.

[32] Management Sciences for Health. International Drug Price Indicator Guide. 2012; Available at: http://erc.msh.org/dmpguide/index.cfm?search_cat=yes&display=yes&module=dmp. Accessed 1/12, 2014.

[33] Jahanzeb M (2008). Adjuvant trastuzumab therapy for HER2-positive breast cancer. Clin Breast Cancer.

[34] Parkinson B, Pearson SA, Viney R (2013). Economic evaluations of trastuzumab in HER2-positive metastatic breast cancer: a systematic review and critique. Eur J Health Econ.

[35] When the price is right. Nat Biotechnol. 2006;24(5):473. 10.1038/nbt0506-473 16680108

[36] Adler MJ, Dimitrov DS (2012). Therapeutic antibodies against cancer. Hematol Oncol Clin North Am.

[37] Rexer BN, Arteaga CL (2012). Intrinsic and acquired resistance to HER2-targeted therapies in HER2 gene-amplified breast cancer: mechanisms and clinical implications. Crit Rev Oncog.

[38] Vargas-Rivas JE, Montes-Casas MM, Cancela-Diez B, Martinez-Martinez F, Sabater-Hernandez D, Calleja-Hernandez MA (2012). Study of compliance with prescription information sheet of trastuzumab prescriptions in a tertiary level hospital. Farm Hosp.

[39] US$54 billion worth of biosimilar patentsexpiring before 2020. 2011; Available at: http://www.gabionline.net/Biosimilars/Research/US-54-billion-worth-of-biosimilar-patents-expiring-before-2020. Accessed 03/28, 2013.

[40] Lozano R, Naghavi M, Foreman K, Lim S, Shibuya K, Aboyans V (2012). Global and regional mortality from 235 causes of death for 20 age groups in 1990 and 2010: a systematic analysis for the Global Burden of Disease Study 2010. Lancet.

[41] Murray CJ, Vos T, Lozano R, Naghavi M, Flaxman AD, Michaud C (2012). Disability-adjusted life years (DALYs) for 291 diseases and injuries in 21 regions, 1990–2010: a systematic analysis for the Global Burden of Disease Study 2010. Lancet.

[42] Gyorki DE, Muyco A, Kushner AL, Brennan MF, Kingham TP (2012). Cancer surgery in low-income countries: an unmet need. Arch Surg.

[43] Eng A, McCormack V, dos-Santos-Silva I (2014). Receptor-defined subtypes of breast cancer in indigenous populations in Africa: a systematic review and meta-analysis. PLoS Med.

[44] Bazargani YT, Ewen M, de Boer A, Leufkens HGM, Mantel-Teeuwisse AK (2014). Essential medicines are more available than other medicines around the globe. PLoS One.

