# Supplementary material for: Essential medicines for breast cancer in low and middle income countries
Source: BMC Cancer. 2015 Aug 18;15:591. doi: 10.1186/s12885-015-1583-4 (PMC4538762; doi:10.1186/s12885-015-1583-4)

Annexes:

Annex 1 - Overview of LMICs included in the study (World Bank income level, WHO region, year of NEML publication)

Country	World bank income level	WHO region	Year of (latest update) Essential Medicines list
Afghanistan	low income	Eastern Mediterranean	2007
Algeria	upper middle income	Africa	2006
Angola	upper middle income	Africa	2008
Armenia	lower middle income	Europe	2010
Bangladesh	low income	South-East Asia	2008
Belize	lower middle income	America	2009
Bhutan	lower middle income	South-East Asia	2009
Bolivia	lower middle income	America	2011
Brazil	upper middle income	America	2010
Burkina Faso	low income	Africa	2007
Burundi	low income	Africa	2009
Cameroon	lower middle income	Africa	2011
Central African Republic	low income	Africa	2009
Chad	low income	Africa	2007
China	upper middle income	Western Pacific	-----*
Congo	lower middle income	Africa	2008
Congo, D.R.	low income	Africa	2010
Cooks Island	NA	Western Pacific	2007
Djibouti	lower middle income	Eastern Mediterranean	2007
Dominic Republic	upper middle income	America	2005
Ecuador	upper middle income	America	2009
Egypt	lower middle income	Eastern Mediterranean	2006
El Salvador	lower middle income	America	2011
Eritrea	low income	Africa	2010
Ethiopia	low income	Africa	2010
Fiji	lower middle income	Western Pacific	2006
Ghana	lower middle income	Africa	2010
Guyana	lower middle income	America	2009
Honduras	lower middle income	America	2009
India	lower middle income	South-East Asia	2011
Indonesia	lower middle income	South-East Asia	2008
Iraq	lower middle income	Eastern Mediterranean	2010
Jamaica	upper middle income	America	2008
Jordan	upper middle income	Eastern Mediterranean	2009
Kenya	low income	Africa	2010

Kiribati	lower middle income	Western Pacific	2009
Kyrgyzstan	low income	Europe	2009
Lesotho	lower middle income	Africa	2005
Madagascar	low income	Africa	2008
Malaysia	upper middle income	Western Pacific	2008
Maldives	upper middle income	South-East Asia	2009
Mali	low income	Africa	2008
Mauritania	low income	Africa	2007
Moldova	lower middle income	Europe	2009
Morocco	lower middle income	Eastern Mediterranean	2008
Namibia	upper middle income	Africa	2008
Nauru	NA	Western Pacific	2010
Nepal	low income	South-East Asia	2009
Nicaragua	lower middle income	America	2011
Nigeria	lower middle income	Africa	2010
Niue	NA	Western Pacific	2006
Pakistan	lower middle income	Eastern Mediterranean	2007
Paraguay	lower middle income	America	2009
Peru	upper middle income	America	2010
Philippines	lower middle income	Western Pacific	2008
Rwanda	low income	Africa	2010
Senegal	lower middle income	Africa	2008
Seychelles	upper middle income	Africa	2010
Solomon Islands	lower middle income	Western Pacific	2010
Somalia	NA	Eastern Mediterranean	2006
South Africa	upper middle income	Africa	2006
Sri Lanka	lower middle income	South-East Asia	2009
St. Vincent and the Grenadines	upper middle income	America	2010
Sudan	lower middle income	Eastern Mediterranean	2007
Tajikistan	low income	Europe	2009
Tanzania	low income	Africa	2007
Thailand	upper middle income	South-East Asia	2008
Tonga	lower middle income	Western Pacific	2007
Tuvalu	upper middle income	Western Pacific	2008
Uganda	low income	Africa	2007
Ukraine	lower middle income	Europe	2009
Uruguay	upper middle income	America	2011
Vanuatu	lower middle income	Western Pacific	2007
Yemen	lower middle income	Eastern Mediterranean	2007
Zimbabwe	low income	Africa	2006

* In China, provincial EMLs were combined to make an EML list, instead of the NEML

Annex2 - International consensus guidelines for breast cancer treatment:

The guideline	Type of cancer	Type of tumor	Main therapy	First line treatment	Second line treatment	Notes
**1st international consensus for advanced breast cancer (2012)**	advanced breast cancer	ER+/HER2-	ET	tamoxifen (LMICs) or AIs (in the rest)*	tamoxifen or AIs or fulvestrant, megestrol acetate	*: for Postmenopausal women
		HER2+	Anti-HER2 agents	trastuzumab or lapatinib	trastuzumab & lapatinib	
				trastuzumab & CT	(trastuzumab & CT*) or (lapatinib & capecitabine)	*:CT as explained in HER2-
		ER+/HER2+	Anti-HER2 agents+ ET	(trastuzumab , lapatinib) & (tamoxifen or AI)	(trastuzumab & lapatinib) & (tamoxifen or AI)	
		HER2-	CT	Anthracycline or taxane based regimens or capecitabine or vinorelbine		Sequential chemotherapy instead of combination chemotherapy
**St Gallen international expert consensus on Primary therapy of Early breast cancer(2011)**	early breast cancer	Luminal A	ET	tamoxifen or AIs*		*: AIs if tamoxifen is contraindicated or Postmenopausal
		Luminal B (HER2-)	ET+/-CT	tamoxifen or AIs*(ET)<br>Both Anthracyclines &Taxanes (CT)		*: AIs if tamoxifen is contraindicated or Postmenopausal
		Luminal B (HER2+)	CT+ ET+ Anti-HER2 agents	tamoxifen or AIs* (ET)<br>Both Anthracyclines & Taxanes(CT)<br>Anti-HER2 agents		*: AIs if tamoxifen is contraindicated or Postmenopausal
		Non luminal (HER2+)	CT+ Anti-HER2 agents	CT+ Anti-HER2 agents		
		Triple negative	CT	Anthracyclines &Taxanes and an alkylating agent (typically cyclophosphamide)		
		Special histological types	ET	tamoxifen or AIs*		*: AIs if tamoxifen is contraindicated or Postmenopausal
			CT	CT		

ER: estrogen receptor; HER2: human epidermal growth factor receptor type 2 oncogene; HER2+: HER2 overexpressed tumor;HER2-: not HER2+; CT: chemotherapy; ET: endocrine therapy;

Annex 3 - Frequency of inclusion of oncology medicines indicated for breast cancer in the NEMLs studied

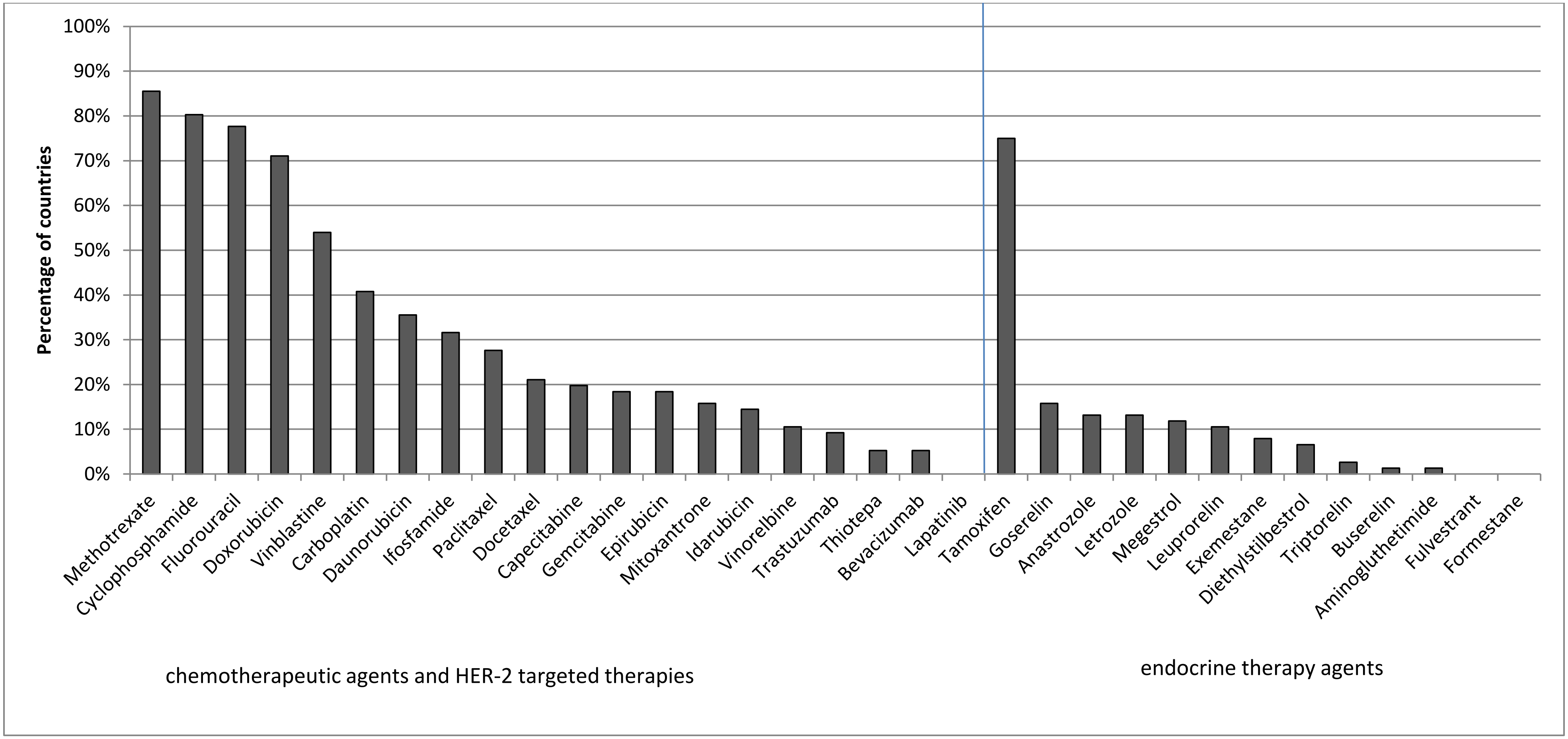

Supplement: Additional file 1: — Annex 1. Overview of LMICs included in the study (World Bank income level, WHO region, year of NEML publication). Annex 2: International consensus guidelines for breast cancer treatment. Annex 3: Frequency of inclusion of oncology medicines indicated for breast cancer in the NEMLs studied. (PDF 449 kb) [file 12885_2015_1583_MOESM1_ESM.pdf]
